# Increased efficiency of direct nanoimprinting on planar and curved bulk titanium through surface modification^☆^

**DOI:** 10.1016/j.mee.2013.05.016

**Published:** 2013-12

**Authors:** Andrew I.M. Greer, Krishna Seunarine, Ali Z. Khokhar, Ian MacLaren, Alistair S. Brydone, David A.J. Moran, Nikolaj Gadegaard

**Affiliations:** a Division of Biomedical Engineering, School of Engineering, University of Glasgow, Glasgow G12 8LT, UK; b School of Physics, University of Glasgow, Glasgow G12 8QQ, UK; c Division of Electronics and Nano-Scale Engineering, School of Engineering, University of Glasgow, Glasgow G12 8LT, UK

**Keywords:** Nanoimprint, Implant, Metal, Stem-cell, Topography

## Abstract

In this work the direct transfer of nanopatterns into titanium is demonstrated. The nanofeatures are imprinted at room temperature using diamond stamps in a single step. We also show that the imprint properties of the titanium surface can be altered by anodisation yielding a significant reduction in the required imprint force for pattern transfer. The anodisation process is also utilised for curved titanium surfaces where a reduced imprint force is preferable to avoid sample deformation and damage. We finally demonstrate that our process can be applied directly to titanium rods.

## Highlights

• Direct nanopatterning of titanium using nanopatterned diamond based stamps.
• Quantify nanopillar matrix imprint depth with regards to feature size and density.
• An account of a novel method of reducing the imprint load required to emboss titanium.
• TEM and EELS analysis following our load reduction treatment.
• The first demonstration of nanopatterning curved, bulk titanium.

## Graphical abstract

**Figure:**
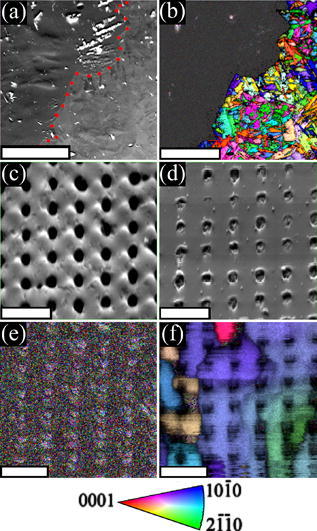

## 1 Introduction

Direct nanopatterning of metal is a technology with applications in electronics and optics where high throughput, low cost and low emission methods for the mass production of circuitry [1], mass storage media [2] and optical devices [3] is concerned. Here the adoption of the technology for the bio-engineering application of implant surface modification is examined.

Dalby et al. have shown that disordered arrays of nanopits may stimulate bone formation from bone marrow stem cells upon an implant’s surface [4]. This work was carried out on PMMA substrates using hot embossing. Thus to translate the original findings to an implant relevant material such as titanium, it is necessary to find an efficient way of introducing such precisely engineered nanotextures onto large areas of curved titanium. There are several methods derived from the microelectronics industry which may be considered for achieving such controlled topographical alterations. Methods such as: chemical etching [5], electrochemical machining [6], electro-discharge machining [7], imprinting [8], laser modification [9], oxidation [10] and reactive-ion-etching (RIE) [11]. Nanopatterning for cell interaction may also benefit from the ability to control both nanofeature anisotropy and aspect ratio. As a result of these prerequisites imprinting, oxidation and laser modification are currently the three favourable approaches. However, laser modification will induce stress gradients and chemical changes to the titanium which is reported to be problematic for load bearing applications and is thus not suitable as an osteologic interface [12]. This paper examines direct nano-imprinting as a method for achieving the goal of creating an efficient titanium implant patterning system and ultimately reports an advancement in the process efficiency through surface oxidation.

## 2 Experimental

During compression, materials deform elastically before inducing permanent plastic deformation. Two seperate parameters are important for analysing a material’s behaviour in these two deformity regions. Resistance to elastic deformation can be quantified by the Young’s modulus (in units of GPa) and resistance to plastic deformation can be quantified by the Knoop hardness (in units of HK). For this reason both the Young’s modulus and Knoop hardness were considered when selecting the stamp material.

The required stamp material needed to be harder than titanium (which has a Knoop hardness of 139 HK [13]) so that it would induce permanent deformation of the titanium and at the same time exhibit low elastic deformation under compression. A low elastic deformation of the stamp during the imprint, enables the titanium to reach its plastic deformation region with negligible distortion of the nanofeatures in the stamp. Further requirements set by the lithographic patterning approach is (1) the availability of the stamp material in a wafer format and (2) the ability to transfer the pattern to the substrate by an etch process. Several materials were considered of which diamond is the material with the highest values of Young’s modulus (1050 GPa [14]) and Knoop hardness (7000 HK [15]); thus the expected to be the ideal candidate for imprinting titanium with the least degradation.

### 2.1 Diamond stamps

**Fig. 1:**
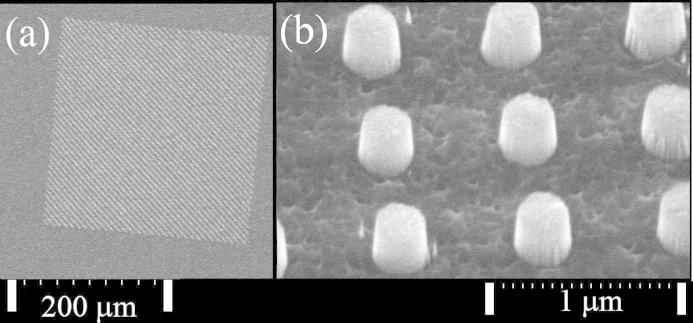
Scanning electron micrograph planar view of: (a) a 250 × 250 μm^2^ array of 200 nm diameter pillars, (b) close-up scanning electron micrograph of 200 nm pillars at 45° tilt.

We used 2 μm of ultra-nanocrystalline diamond (UNCD) grown by chemical vapour deposition (CVD) on a ∼520 μm silicon carrier wafer from Advanced Diamond Technologies Ltd. Detailed information about the material and the stamp fabrication can be found in our earlier paper [16]. The UNCD wafer was scribed into 1 × 1 cm^2^ samples and subjected to RCA cleaning (SC-1), followed by ultrasonic solvent cleaning. Nanofeature stamps were then created from the samples using conventional electron beam lithography (EBL) with negative tone electron sensitive resist, hydrogen silsesquioxane (HSQ). An Al discharge layer was required above the resist to prevent e-beam deflection due to charge build-up on the surface [17]. Several stamps were produced with this process and the pattern written varied in design but consisted of arrays of circular pillars. After EBL and HSQ development, the HSQ was used as an etch mask for RIE with a mixture of oxygen and argon gas. The etched diamond nanopillars were typically 225 nm high. Fig. 1 displays a scanning electron micrograph of some typical stamp features.

### 2.2 Titanium substrates

In order to explore the capabilities of direct nano-imprinting, two different forms of titanium substrate were prepared: (1) discs and (2) rods. Both substrates were cut from the same commercially pure 10 mm diameter titanium rods (supplied by Righton Ltd.).

(1) The discs were cut from the stock rod with a thickness of 6 mm. One face of each of the discs was polished using a seven stage chemical mechanical polishing process on a Buehler MotoPol 2000 polishing machine. The first four stages used increasingly finer grades (220, 500, 800 and 1200) of wet SiC grit paper. The three subsequent stages used a soft Kemet Chem-H polishing pad with 1 μm diamond slurry and colloidal silica for more prolonged polishing to remove the micro-scratches remaining from previous abrasive stages.^1^ The arithmetic average (Ra) titanium roughness achieved after the final polish stage was measured by AFM (over a scan area of 25 × 25 μm) to be <3 nm.^1^
(2) Longer sections of the rod (∼4 cm in length) were cut and prepared with the same grinding and polishing stages as for the discs. In this case an AFM was used along a 25 × 2 μm length of the rod and yielded an arithmetic average roughness of <18 nm.

### 2.3 Titanium anodisation

Anodisation is an electrolytic passivation process that is used to increase the thickness of the native oxide layer on metal parts, such as titanium. An electrolyte consisting of 0.3 M oxalic acid and reverse osmosis water was continually stirred, while being maintained at a temperature of ∼17 °C. A platinum wire mesh (supplied by Goodfellow Cambridge Ltd.) was used as a cathode and the titanium work piece to be anodised acted as an anode. The anode voltage was slowly ramped up from 0 V to the desired level over a period of approximately 5 min. After anodisation the titanium discs were removed from the electrolyte and thoroughly rinsed in RO water before being dried in a nitrogen stream.

### 2.4 Imprinting

Direct imprinting of titanium was performed using a manual hydraulic press supplied by Specac Ltd. The stamp and titanium work piece were mounted between two 5 × 25 × 25 mm tungsten carbide/cobalt sheets (supplied by Goodfellow Cambridge Ltd.) to allow the load to be distributed over a larger area, thus preventing sinking into the underlying hydraulic press plates. Loads between 100 and 800 kg were applied with a hold time of 30 s for each load.

## 3 Results and discussion

**Fig. 2:**
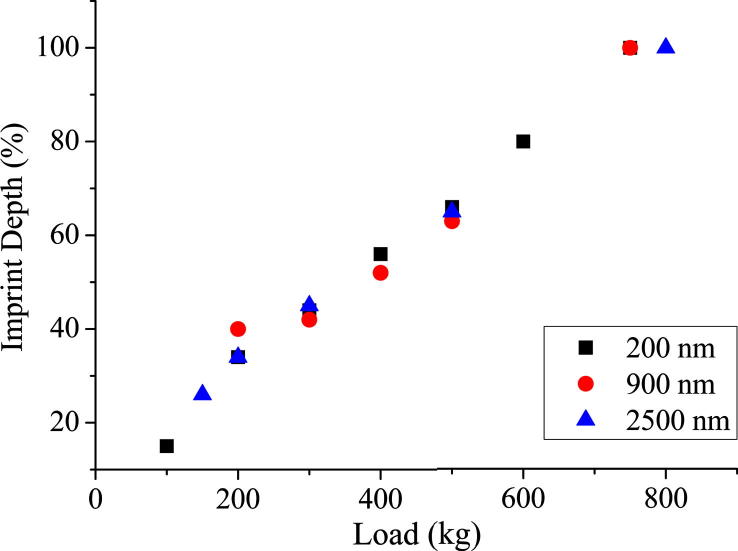
Plot of imprint depth into planar Ti (measured by AFM) against imprint load for 200, 900 and 2500 nm pillar matrixes with the same contact area (6250 μm^2^).

**Fig. 3:**
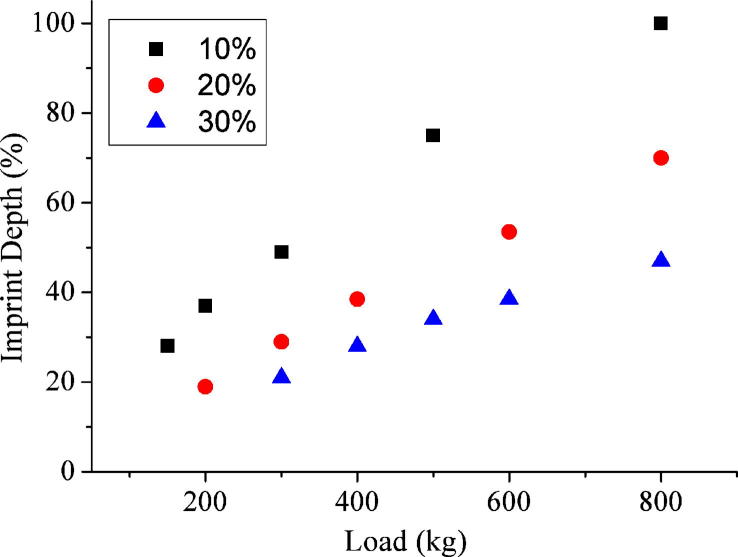
Plot of imprint depth into planar Ti (measured by AFM) against imprint load for 2500 nm pillar matrixes for 10% (6250 μm^2^), 20% (12,500 μm^2^) and 30% (18,750 μm^2^) feature density (contact area with Ti surface).

Imprinting of titanium discs were carried out using diamond stamps and the imprint depth was measured by AFM. It was observed that the contact area, as expected, had a more profound effect on imprint depth than feature size, as can be seen from Figs. 2 and 3. Fig. 2 shows imprint depth (as measured by AFM) against pressing load for 200, 900 and 2500 nm diameter pillar stamps with constant feature face area of 10% into the titanium discs. This plot illustrates that all three feature sizes imprint to a comparable depth when the contact area is kept constant. Fig. 3 displays the variation in imprint depth against the imprint load for three 2500 nm diameter pillar stamps, each with a different feature density: 10%, 20% and 30% into the titanium discs. This plot also indicates that imprint depth is pressure dependent as stamps with increased contact areas require proportionally larger pressing loads to achieve the same depth of imprint.

### 3.1 Surface modification by anodisation

**Fig. 4:**
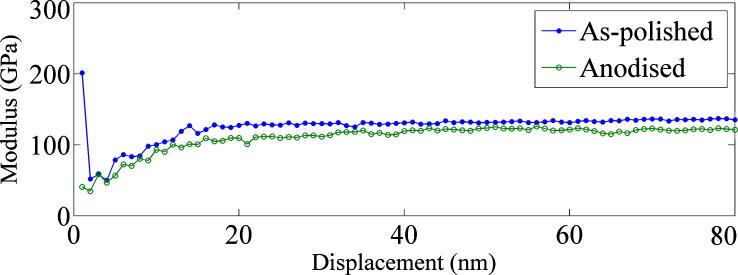
Plot of average Young’s modulus measured by dynamic nanoindentation for the as-polished samples (blue solid circles) and anodised samples (green open circles) against imprint depth. (For interpretation of the references to colour in this figure legend, the reader is referred to the web version of this article.)

As with most metals, titanium forms a natural oxide layer at the surface when exposed to air [18]. The native titanium oxide is reported to be beneficial for bio-integration as it acts as a barrier for ionic leakage [19]. For some applications the thickness of oxide layer is increased which leads to a colour change due to optical interference. This effect can be used to provide a clear visual identification of parts yet maintaining the interfacial characteristics for implant-tissue interaction. Blau [20] reported that the direct imprint characteristics of metals are affected by the presence of such an oxide layer. In order to determine the effect the oxide layer has on the imprint process, comparative dynamic nanoindentation analysis was performed on both an anodised sample and an as-polished sample using a Berkovich tip nanoindentation machine.^1^ Fig. 4 displays the average resultant Young’s moduli with regard to imprint depth during the nanoindentation processing (each plot comprises an average from four discrete indents). It is visible from Fig. 4 that the initial modulus is around four times higher for as-polished samples. Thereafter an offset of around 10 GPa remains between the anodised and as-polished samples, with the anodised version exhibiting the lower level of stiffness. Although only the surface has been modified, due to the geometry of a Berkovich tip (three-sided triangular pyramid) the surface is constantly contributing to the effective modulus experienced by the indenter which is why the offset between the two moduli remains constant despite the tip displacement exceeding the surface oxide depth.

**Fig. 5:**
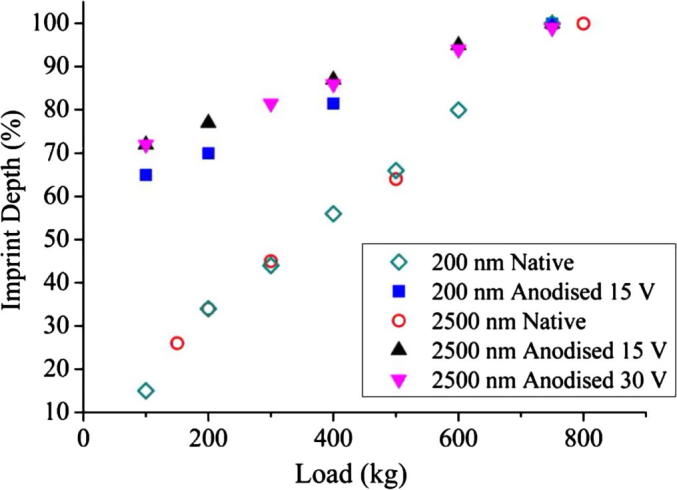
Plot of imprint depth (measured by AFM) against imprint load for stamps with a fixed feature density (contact area) 10% (6250 μm^2^) into as-polished Ti (open markers) and anodised Ti (filled markers).

Following the indication from the nanoindentation analysis that stiffness may be reduced through anodising, imprinting was repeated on Ti samples with thicker anodic oxide layers. Fig. 5 shows that the imprint depth (as measured by AFM) of the anodised samples is sizably deeper than the as-polished samples for most of the tested data points. Fig. 5 also shows that the imprint depth is similar for both of the anodised surfaces despite one of them being anodised with twice the potential. This suggests that the process of anodising induces a hardness decrease that is independent of the oxide thickness for the tested range. The results were similar for both 2500 and 200 nm features covering the same contact area, so the perceived softening is not dependent on feature size.

The concept of anodisation reversing the effects of work-hardening induced by the polishing stage is discredited by literature. Hunt et al. [21] show through experimentation that no work hardening is induced in titanium by means of chemical mechanical polishing and Kim et al. [22] report that the diffusion of oxygen into titanium results in surface hardening rather than softening.

### 3.2 Electron backscatter diffraction

**Fig. 6:**
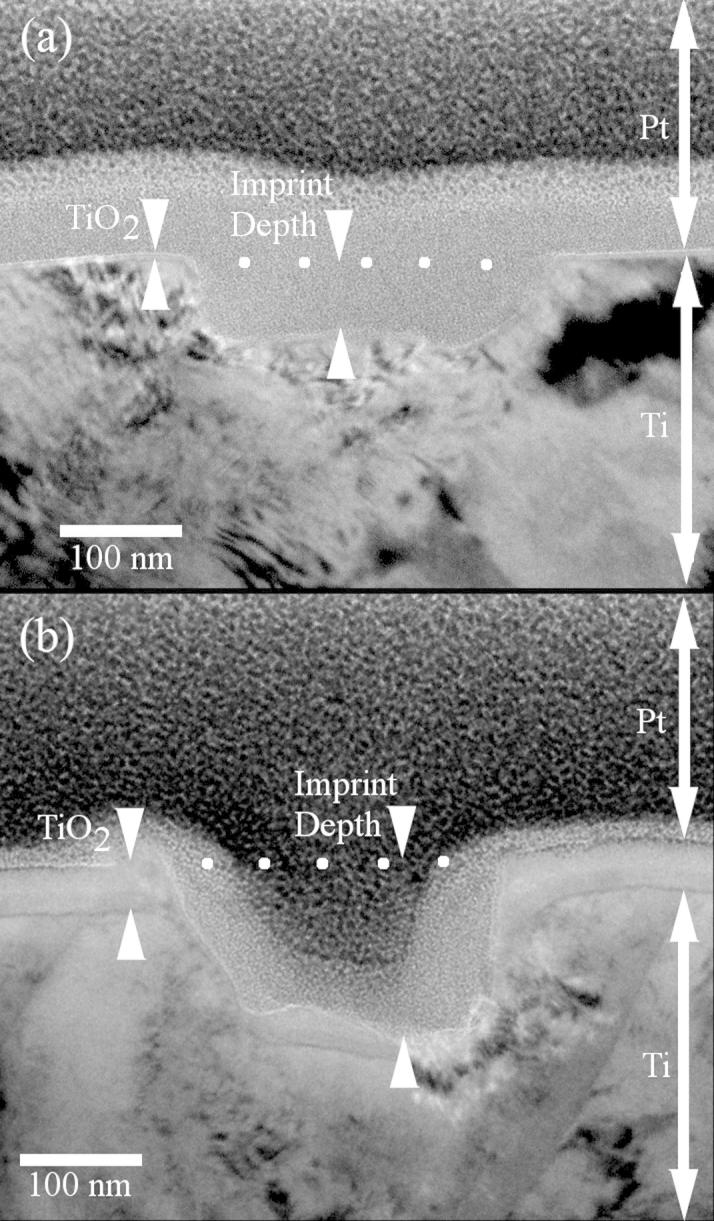
(a) SEM micrograph of the boundary (indicated by a super imposed dotted red line) between an anodised section (left of the dotted red line) and an as-polished section (right of the dotted red line) of a Ti substrate. Scale bar = 60 μm. (b) Alpha-Ti inverse pole figure map of (a) overlaid on top of (a). Scale bar = 60 μm. (c) SEM micrograph of a 200 nm, 10% feature density, 100 kg imprint into anodised Ti. Scale bar = 1 μm. (d) SEM micrograph of a 200 nm, 10% feature density, 100 kg imprint into as-polished Ti. Scale bar = 1 μm. (e) Alpha-Ti inverse pole figure map of (c) overlaid on top of (c). Scale bar = 1 μm. (f) Alpha-Ti inverse pole figure map of (d) overlaid on top of (d). Scale bar = 1 μm. (b), (e) and (f) use the colour key at the bottom of the figure. (For interpretation of the references to colour in this figure legend, the reader is referred to the web version of this article.)

Electron backscatter diffraction (EBSD) was carried out using an FEI Quanta 200F SEM to analyse the two different surfaces.^1^ It was determined during the processing of the EBSD data with EDAX Microanalysis software that the anodised surface was completely amorphous. Fig. 6(a) displays an SEM image of the boundary between an anodised area on the left and an as-polished area on the right. Fig. 6(b) is Fig. 6(a) with its inverse pole figure map overlaid. Fig. 6(b) shows clearly that the as-polished area produces clean diffraction patterns of the underlying grains whereas the anodised side appears exempt from a diffraction pattern due to the non-crystalline structure of the surface. Analysis of imprinted areas is also shown in Fig. 6(c) and (d) are SEM micrographs of the anodised and as-polished imprints, respectively and Fig. 6(e) and (f) contain the overlaid inverse pole maps of Fig. 6(c) and (d), respectively. The imprints under analysis in Fig. 6(c–f) are those produced by 200 nm diameter, 10% feature density stamps after embossing with a 100 kg load. Fig. 6(d) shows partial and shallow non-uniform imprinting whereas Fig. 6(c) appears deeper and more uniform. In general imprints possessed a radial gradient of imprint depth so misorientation maps of imprinted 200 nm pillar matrices on as-polished Ti were also examined.^1^ These plots indicated that dislocation density increases towards the centre of the area being imprinted. This increase in dislocation density is known as work hardening and is evidently induced by the imprint process.

### 3.3 Transmission electron microscopy

**Fig. 7:**
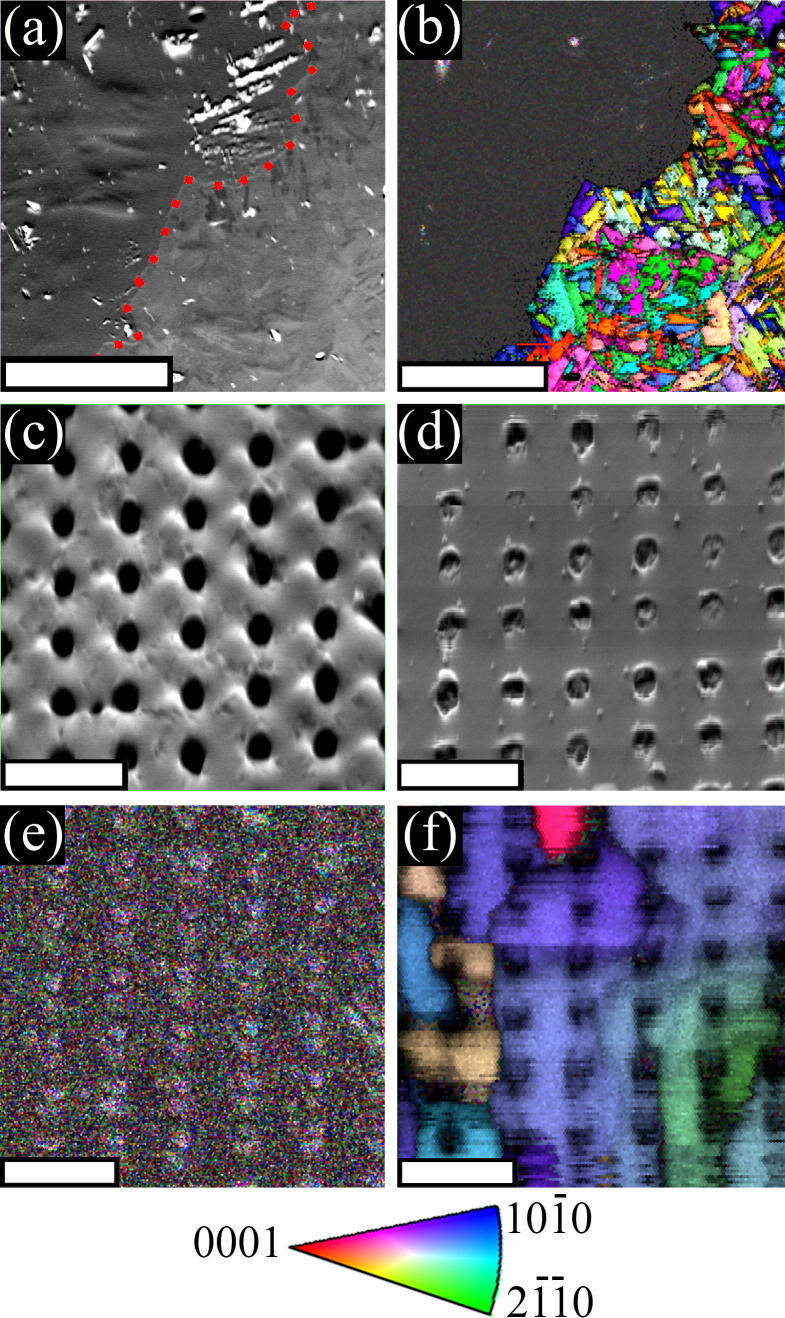
Transmission electron micrograph showing cross-sections of feature imprints into Ti using a 200 nm, 10% feature density stamp and 100 kg load: (a) Sample with 5 nm native oxide; (b) Sample with 40 nm anodic oxide. In both cases, white dots indicate original Ti surface position. Pt was deposited onto the surfaces in FIB preparation to protect the surface from ion beam damage.

Transmission electron microscopy (TEM) was performed on cross-sections of both anodised and as-polished imprinted titanium samples in order to investigate the surface properties before and after anodising. Small, electron-transparent, cross sections through regions containing a number of trenches were prepared using a focused Ga^+^ ion beam lift-out technique (Nova 200 Dualbeam FIB, FEI, Eindhoven, Netherlands). Samples were prepared from both pre- and post-anodised titanium substrates imprinted with 200 nm diameter features. TEM was then used to examine the crystalline structure of the layers that made up the cross-section. Fig. 7(a) displays a transmission electron micrograph of a cross-section for an individual feature imprint site on as-polished titanium and Fig. 7(b) displays a comparable micrograph for the anodised version.

TEM results confirmed that the native oxide layer on the as-polished titanium substrate was approximately 5 nm thick, while the oxide thickness on the anodised titanium substrate was approximately 40 nm thick. TEM dark field imaging showed that both native and anodised layers contained nanocrystals at the boundary between the surface oxide and underlying Ti.^1^ The nanocrystals in the native oxide (above the as-polished samples) consumed the entire depth of the 5 nm layer, whereas the crystals in the anodic oxide were relatively small in comparison to the 40 nm oxide depth. Electron Energy Loss Spectroscopy (EELS) was performed on both substrates^1^ and the shape of the Ti-L_2,3_ edges was clearly consistent in both cases with TiO_2_ in the anatase polymorph. The majority of the 40 nm anodic oxide layer was determined to be amorphous TiO_2_.

Pethica et al. [23] reported a 5 nm thick native oxide layer on Ni to be responsible for increasing the hardness of Ni beyond that of the bulk material by a factor of 10. Nix [24] reported that thin films above substrates exhibit higher strengths than their bulk counterparts and attributes this to two main factors. The first is that thin films typically have small grains and as Narutani et al. [25] explain dislocation density increases with the reciprocal of grain size so thin films inherently exhibit more strength due to the quantity of dislocations. Secondly, the presence of the substrate acts as a barrier to constrain the dislocations and maintain a dense volume in the surface oxide layer. Nix [24] also provides equations for calculating the bi-axial stress required to move dislocations in thin films and highlights the fact that yield strength is inversely dependent on film thickness.

We believe that our findings are in agreement with the above literature. We propose that the decrease in perceived hardness witnessed in our work stems from the fact that the surface oxide layer thickness is increased beyond the native 5 nm through anodising which decreases the dislocation density through the growth of an amorphous extension. Dislocations therefore experience less inhibited movement thus the hardness of the material is reduced.

### 3.4 Imprinting on curved surfaces

The majority of titanium implants are not planar, many are cylindrical. This naturally provides a challenge for transferring the nanopatterns at the loads required without damaging or distorting the implant. Hence the development of the anodisation step to significantly lower the required imprint force offers a real solution to this problem. In order to illustrate that direct imprinting may be applied to a curved surface, a titanium rod with a diameter of 10 mm and length of 50 mm was prepared and polished as described in Section 2.2. The rods were anodised at 15 V potential, using the process described in Section 2.3. The pattern transfer was then released by rolling the polished rod over a nanopatterned stamp. This method can be considered the inverse of conventional roller embossing, where a patterned rod is rolled over a planar substrate.

Diamond stamps were specifically designed for this experiment. Two designs were used. The first design featured four 2.5 mm by 0.25 mm pillar matrixes in parallel. The pillars covered 10% of each matrix area, each of the four matrixes were composed of a different diameter of pillar: 1 μm, 600 nm, 250 nm and 190 nm. The etch time for this stamp was reduced to create shallower features, of 160 nm, for extra robustness. The second design was a 5 × 5 mm^2^ array of disordered 100 nm diameter pillars featuring the same disordered positioning as the nanopits created by Dalby et al. to stimulae bone formation [4]. These pillars were also 160 nm tall.

Since the stamp contact area is a tangential arc when roller imprinting, the applied load does not need to be on the same scale as the planar imprint work discussed in the experimental section. A polished, anodised rod was rolled over all four of the UNCD feature matrixes simultaneously with a load of 3.5 kg. The result was an imprint of all four matrixes on a continuous 2.5 mm length of the rod circumference and shown by AFM to be 50 nm deep. The matrixes were not uniformly embossed at this load, only the edge features of each matrix were imprinted to 50 nm.

**Fig. 8:**
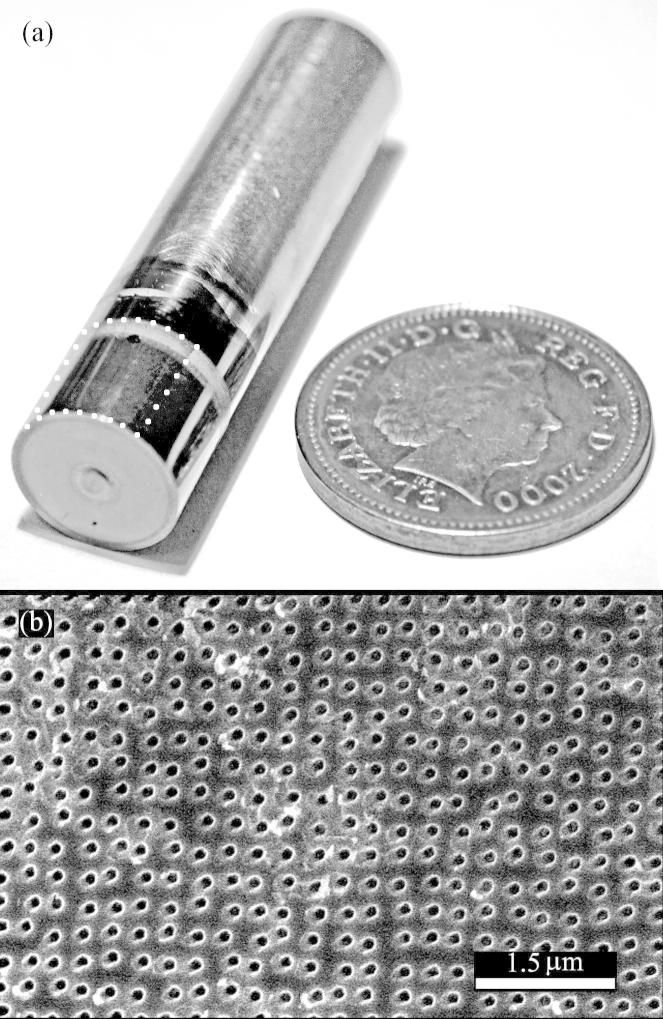
(a) a photograph of a partially polished and anodised 4 cm long 10 mm diameter Ti rod next to a British 2 pence sterling coin. The white dots on the rod indicate the stamped area. (b) An SEM image of the imprinted bioactive 100 nm pits on the surface of the rod shown in image ‘(a)’.

For the second design thicker diamond was sourced in order to facilitate the larger load required to imprint features in the central area of the stamp. Polycrystalline diamond with grain size 50 nm and sample thickness 580 μm was acquired from Element Six. A polished anodised rod was rolled over the stamp at 100 kg. Features were successfully transferred along a 5 mm length of the rod’s circumference (Fig. 8 displays a rod and an SEM image of the transferred bioactive features). All the contact features embossed the rod’s surface but a gradient in imprint depth between perimeter features and central area stamp features remained.

### 3.5 Pattern transfer effects

During imprinting, the first features to plastically deform the surface were those around the perimeter of the stamp (regardless of stamp size). Pei et al. [26] modelled the effect of imprinting a matrix of pillars into metal and concluded that the interaction of stress fields from each pillar would ‘work harden’ the surface. This model explains the radial nature of the non-uniform imprinting; features around the perimeter of the stamp are subjected to less stress fields than those in the more central areas thus the impact of work hardening is greater in the middle of the stamping area. This hypothesis was enforced by the EBSD analysis where misorientation maps indicated an increase in misorientation level towards the centre of the stamped area.^1^ Misorientations are known to be indicative themselves of the level of dislocations present [27] and dislocation accumulation is by definition work hardening.

The Young’s modulus of a material quantifies the material’s resistance to elastic deformation. Titanium has a relatively low value of Young’s modulus when compared to its hardness. This means that the elastic recovery is small. This effect had negligible impact on the diameter of planar imprints however it did affect some of the curved imprint work. The initial imprinted curved titanium features were observed to be up to 50 nm narrower than the stamp features. The amplified effect of elastic recovery on the curved substrate may be attributed to the fact that initial rods were imprinted in one continuous movement with no significant hold time endured. Hold time is important when imprinting metal in order to diminish the effects of both creep and stress relaxation reverse plasticity [28,29]. Elastic recovery was not observed for samples roller embossed at speeds below 0.2 mm/s.

## 4 Conclusions

Direct imprinting offers distinct advantages over other reported methods of introducing precision engineered patterns onto the surfaces of titanium. Direct imprinting is a one-step process that does not introduce any contamination to the sample; however, the imprint forces required to produce patterns over large areas may be too high for practical purposes. With this in mind we have demonstrated a novel way of dramatically reducing the press load forces required for direct nanoimprinting of titanium surfaces, by using anodisation to increase the TiO_2_ layer thickness. It is suggested that thin native TiO_2_ layers inhibit movement of dislocations. However, by increasing the TiO_2_ layer thickness through the addition of amorphous titania via anodisation, dislocation density can be reduced and thus the propagation of dislocations improved. We further demonstrated imprinting of non-planar surfaces. Rods were anodised in the same way as planar titanium work pieces, and then rolled over a diamond stamp. Unlike imprinting planar surfaces, roller imprinting occurred progressively as the rod rolled across the linear contact area. This meant that a much lower overall force was needed, since only a small contact area was imprinted at a time. As a result of both the anodisation and small contact area, imprinting of UNCD nano-pillars was achieved at a load of 3.5 kg. It is advised that roller imprinting is moderately paced in order to allow a hold period to combat the effects of elastic recovery and stress relaxation.

## Appendix A Supplementary data

Supplementary data This document file contains Supplementary Figs. A1–A8.

## Footnotes

1 Electronic Supplementary Information (ESI) available: Ti polishing parameters; Ti AFM roughness scan; EELS plots; TEM dark field images; Nanoindentation plots; EBSD kernel average misorientation maps.
