# Supplementary material for: Increased efficiency of direct nanoimprinting on planar and curved bulk titanium through surface modification
Source: Microelectron Eng. 2013 Dec;112:67–73. doi: 10.1016/j.mee.2013.05.016 (PMC3990420; doi:10.1016/j.mee.2013.05.016)
Supplement: Supplementary data — This document file contains Supplementary Figs. A1–A8. [file mmc1.docx]

Direct nanoimprinting of bioactive features in planar and curved titanium

Supplementary information

Andrew I. M. Greer^a,c^, Krishna Seunarine^a^, Ali Z. Khokhar^a^, Ian MacLaren^b^, Alistair S. Brydone^a^, David Moran^c^ and Nikolaj Gadegaard^a,*^

^a^ Division of Biomedical Engineering, School of Engineering, University of Glasgow, Glasgow, G12 8LT, UK .

^b^ School of Physics, University of Glasgow, Glasgow, G12 8QQ, UK.

c Division of Electronics and Nano-scale Engineering, School of Engineering, University of Glasgow, Glasgow, G12 8LT, UK

^*^Corresponding author (nikolaj.gadegaard@glasgow.ac.uk).

**Sub 3 nm titanium polish parameters for Buhler MotoPol 2000**

| **Pad** | **Table RPM** | **pressure** | **Table direction** | **duration** | **Abrasive /lubricant** |
| --- | --- | --- | --- | --- | --- |
| 220 SiC | 200 | 30N | Counter clockwise | Until flat (10min) | Running H_2_O |
| 500 SiC | 200 | 40N | Counter clockwise | 10min | Running H_2_O |
| 800 SiC | 200 | 40N | Counter clockwise | 10min | Running H_2_O |
| 1200 SiC | 200 | 40N | Counter clockwise | 10min | Running H_2_O |
| Chem-H (Kemet Int. Soft cloth) | 125 | 30N | Counter clockwise | 20min | 10ml of 1um diamond slurry and dripping H_2_O |
| Chem-H (Kemet Int. Soft cloth) | 60 | 50N | Counter clockwise | 60min | 10ml of colloidal silica |
| Chem-H (Kemet Int. Soft cloth) | 60 | 10N | Counter clockwise | 40min (any longer and pitting will occur) | 7 ml colloidal silica and 3 ml 3% hydrogen peroxide |

**Figure A1** Table of parameters in sequential order from top to bottom for polishing commercially pure titanium to sub 3 nm Ra roughness on a Buhler MotoPol 2000 chemical mechanical grinding and polishing machine.


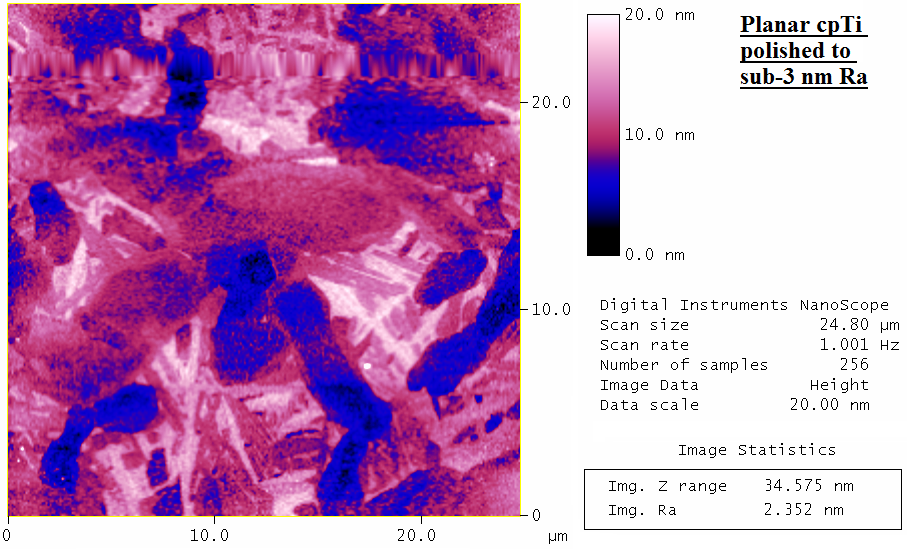


Figure A2 AFM roughness scan of a planar Ti sample after completing the polishing routine. It can be seen that sub-3 nm Ra was achieved.


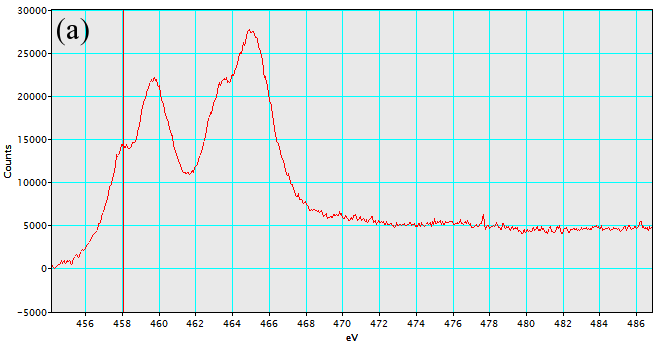


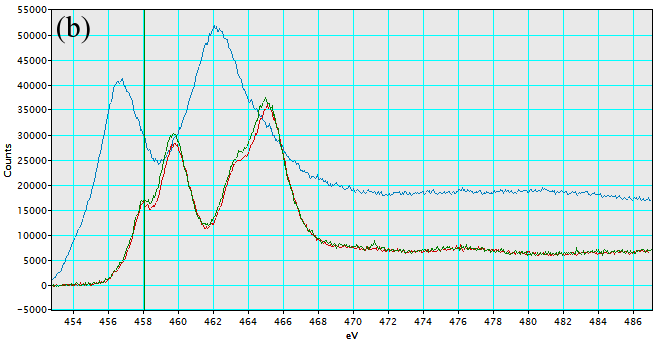
Figure A3 Background subtracted spectra from EELS analysis: a) Native oxide; b) Anodic oxide where the blue trace is the metal, and the red and green traces are from the inner and outer layer in the oxide. In both cases, the oxide trace shows the splitting that would be expected for anatase, but the peaks are broad and the splitting not well defined, suggesting the presence of a significant fraction of amorphous TiO_2_ [32]. A crystalline TiO_2_ standard examined in the microscope on the same day showed a clear splitting of peaks, demonstrating that this broadening was a sample effect and not a consequence of microscope or spectrometer setup.


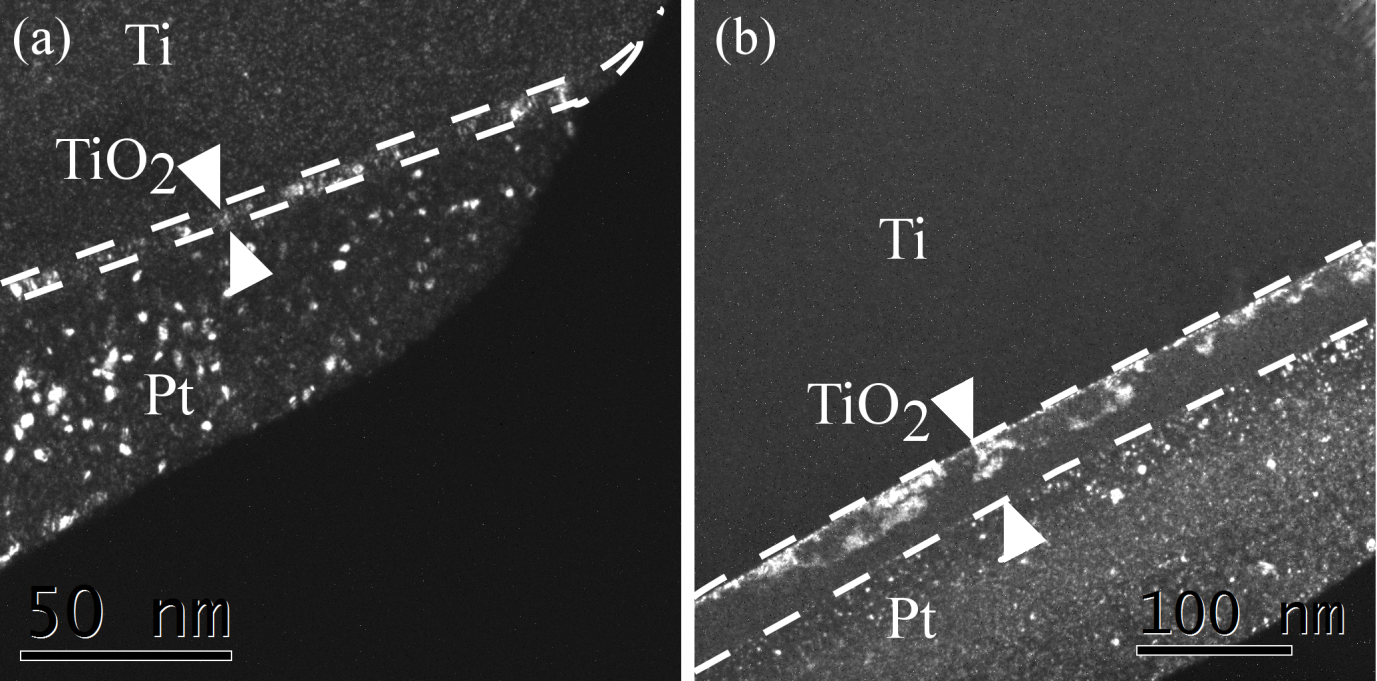


Figure A4 (a) TEM dark field image of native oxide (b) TEM dark field image of 15V anodic oxide. In both images white dashed lines indicate the oxide boundary and the white areas between the lines are reflections of crystals. It can be observed that the nanocrystals consume the entire depth of the native fim and that the anodic film is largely amorphous.


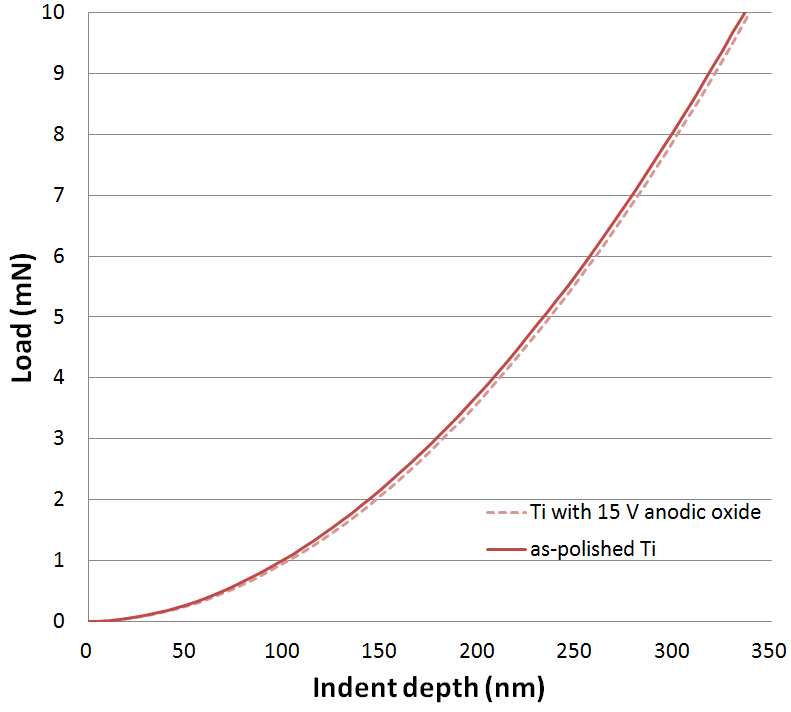


**Figure A5** Dynamic nanoindentation plot of average indentation depth against load from 4 discrete indents per sample into an as-polished sample with ~3 nm oxide (solid line) and an anodised sample with ~ 40 nm oxide (dashed line).


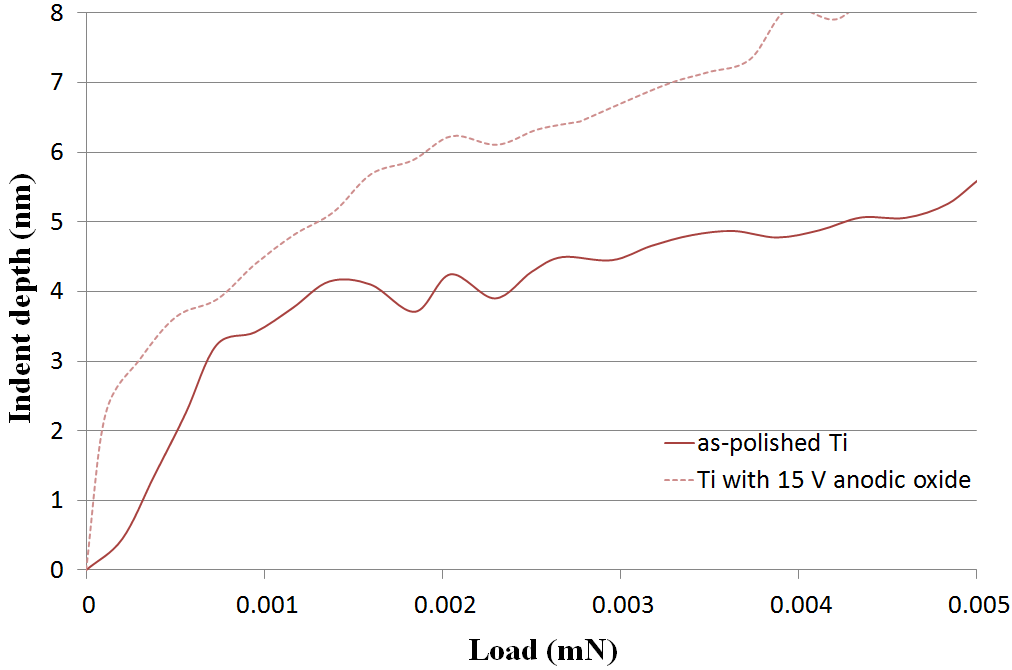


**Figure A6** Dynamic nanoindentation plot of indentation depth against load for two typical indents into an as-polished sample with ~3 nm oxide (solid line) and an anodised sample with ~ 40 nm oxide (dashed line) over the first 8 nm of imprint depth.


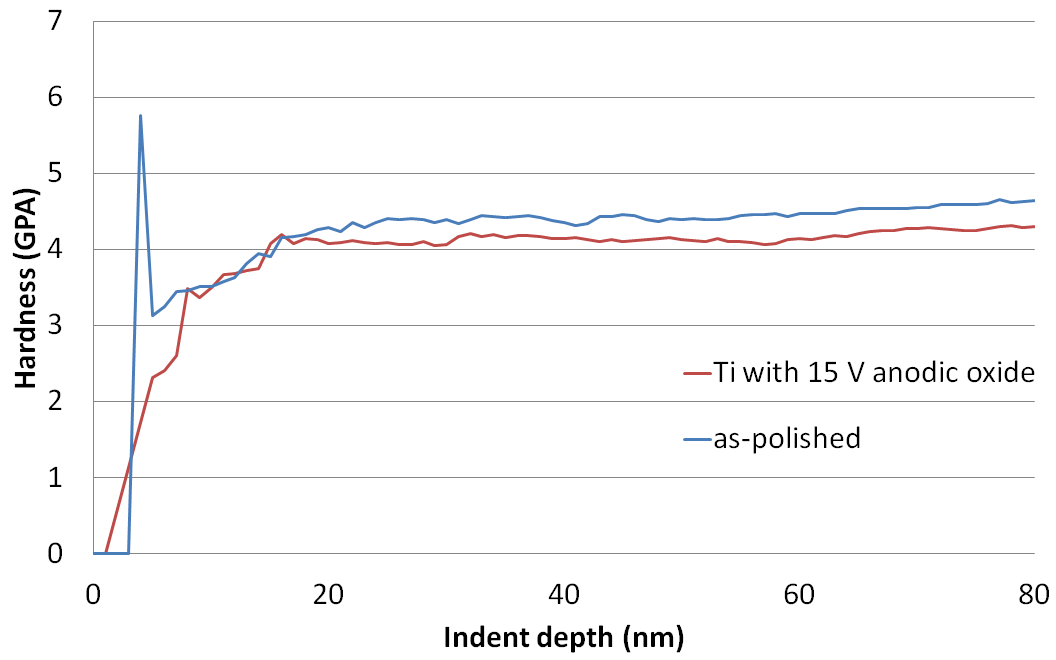


**Figure A7** Average hardness against indent depth determined from four dynamic nanoindentations per sample for an as-polished sample with ~3 nm oxide (blue line) and an anodised sample with ~ 40 nm oxide (red line).


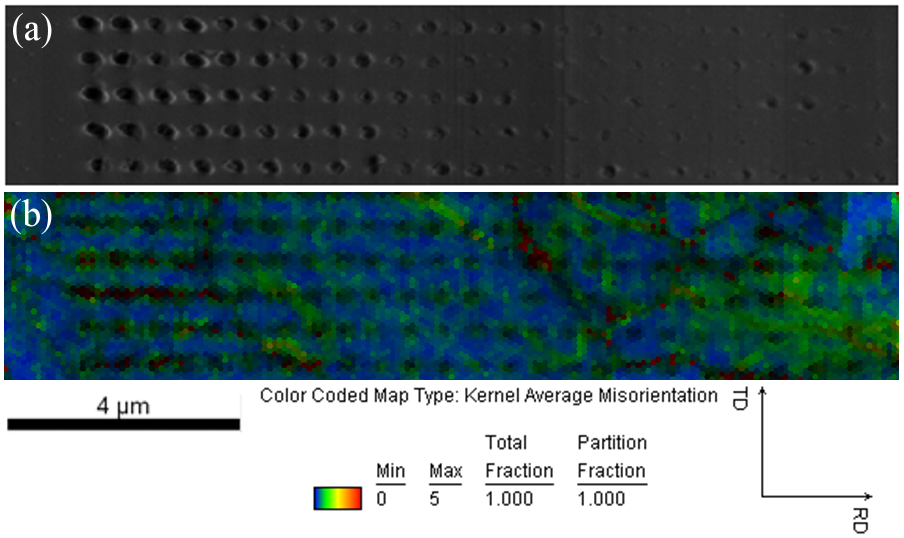


**Figure A8** (a) SEM micrograph of a radially non-uniform 100 kg imprint of a 200 nm diameter, 10% feature density stamp into as-polished Ti. The left side is the outer edge of the imprint. (b) the Kernal Average Misorientation colour coded map of Figure A5 (a). It is observed that within a small distance of 12 microns the imprint depth reduces as the level of misorientations increases. It is proposed that the interaction of stress fields are responsible for this effect which is indicative of dislocation accumulation otherwise known as work hardening induced by the imprint process.
